# Glycozolidal

**DOI:** 10.1107/S1600536811024160

**Published:** 2011-06-25

**Authors:** Hoong-Kun Fun, Wisanu Maneerat, Surat Laphookhieo, Suchada Chantrapromma

**Affiliations:** a X-ray Crystallography Unit, School of Physics, Universiti Sains Malaysia, 11800 USM, Penang, Malaysia; b Natural Products Research Laboratory, School of Science, Mae Fah Luang University, Tasud, Muang Chiang Rai 57100, Thailand; c Crystal Materials Research Unit, Department of Chemistry, Faculty of Science, Prince of Songkla University, Hat-Yai, Songkhla 90112, Thailand

## Abstract

The title compound known as glycozolidal (systematic name: 2,7-dimeth­oxy-9*H*-carbazole-3-carbaldehyde), C_15_H_13_NO_3_, is a naturally occurring carbazole, which was isolated from the roots of *Clausena lansium*. The carbazole ring system is essentially planar, with an r.m.s. deviation of 0.0093 (1) Å. In the crystal, inter­molecular N—H⋯O hydrogen bonds connect the mol­ecules into a chain along the *c* axis. C—H⋯O, C—H⋯π and π–π inter­actions, with centroid–centroid distances of 3.5924 (6), 3.6576 (6) and 3.8613 (6) Å, are also observed.

## Related literature

For bond-length data, see: Allen *et al.* (1987 ▶). For background to carbazole alkaloids and their activities, see: Kongkathip & Kongkathip (2009 ▶); Laphookhieo *et al.* (2009 ▶); Li *et al.* (1991 ▶); Maneerat & Laphookhieo (2010 ▶); Maneerat *et al.* (2010 ▶); Sripisut & Laphookhieo (2010 ▶); Tangyuenyongwatthana *et al.* (1992 ▶); Thongthoom *et al.* (2010 ▶); Yenjai *et al.* (2000 ▶). For related structures, see: Fun *et al.* (2007 ▶, 2009 ▶, 2010 ▶). For the stability of the temperature controller used in the data collection, see: Cosier & Glazer (1986 ▶).
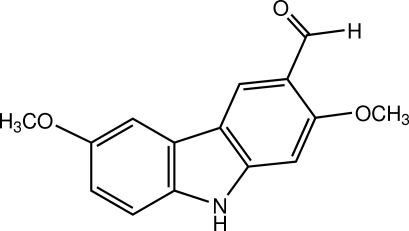


## Experimental

### 

#### Crystal data

C_15_H_13_NO_3_
*M* *_r_* = 255.26
Monoclinic, 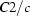

*a* = 20.5756 (4) Å
*b* = 8.1298 (1) Å
*c* = 14.0411 (3) Å
β = 98.871 (1)°
*V* = 2320.64 (7) Å^3^
*Z* = 8
Mo *K*α radiation
μ = 0.10 mm^−1^
*T* = 100 K
0.53 × 0.42 × 0.16 mm

#### Data collection

Bruker APEXII CCD area-detector diffractometer
Absorption correction: multi-scan (*SADABS*; Bruker, 2005 ▶) *T*_min_ = 0.947, *T*_max_ = 0.984
13003 measured reflections
3381 independent reflections
3032 reflections with *I* > 2σ(*I*)
*R*_int_ = 0.020

#### Refinement

*R*[*F* ^2^ > 2σ(*F* ^2^)] = 0.039
*wR*(*F* ^2^) = 0.115
*S* = 1.04
3381 reflections
178 parameters
H atoms treated by a mixture of independent and constrained refinement
Δρ_max_ = 0.45 e Å^−3^
Δρ_min_ = −0.24 e Å^−3^

### 

Data collection: *APEX2* (Bruker, 2005 ▶); cell refinement: *SAINT* (Bruker, 2005 ▶); data reduction: *SAINT*; program(s) used to solve structure: *SHELXTL* (Sheldrick, 2008 ▶); program(s) used to refine structure: *SHELXTL*; molecular graphics: *SHELXTL*; software used to prepare material for publication: *SHELXTL* and *PLATON* (Spek, 2009 ▶).

## Supplementary Material

Crystal structure: contains datablock(s) global, I. DOI: 10.1107/S1600536811024160/is2736sup1.cif

Structure factors: contains datablock(s) I. DOI: 10.1107/S1600536811024160/is2736Isup2.hkl

Supplementary material file. DOI: 10.1107/S1600536811024160/is2736Isup3.cml

Additional supplementary materials:  crystallographic information; 3D view; checkCIF report

## Figures and Tables

**Table 1:** Hydrogen-bond geometry (Å, °) *Cg*2 is the centroid of the C1–C4/C11/C12 ring.

*D*—H⋯*A*	*D*—H	H⋯*A*	*D*⋯*A*	*D*—H⋯*A*
N1—H1*N*1⋯O3^i^	0.890 (17)	2.106 (17)	2.9758 (11)	165.2 (15)
C15—H15*C*⋯O2^ii^	0.98	2.44	3.3888 (14)	162
C15—H15*A*⋯*Cg*2^iii^	0.98	2.91	3.6613 (12)	134

Symmetry codes: (i) 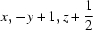; (ii) 
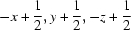
; (iii) 
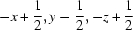
.

**Fig. 1.**
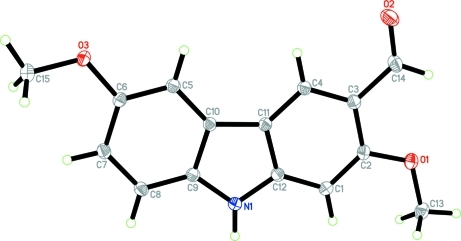
The molecular structure of the title compound, showing 50% probability displacement ellipsoids and the atom-numbering scheme.

**Fig. 2.**
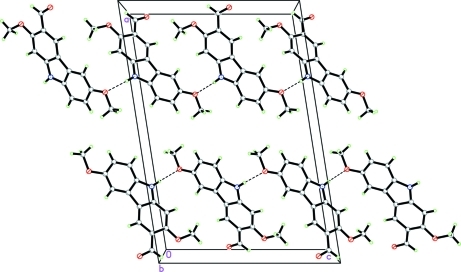
The crystal packing of the title compound viewed along the b axis, showing one dimensional chains along the [0 0 1] direction. Hydrogen bonds are shown as dashed lines.

## supplementary crystallographic information

### Comment

Carbazole alkaloids are major compounds found in Rutaceae plants, especially in *Clausena* genus (Laphookhieo *et al.*, 2009; Li *et al.*, 1991; Maneerat *et al.*, 2010; Sripisut & Laphookhieo, 2010; Tangyuenyongwatthana *et al.*, 1992) which showed diverse pharmacological activities such as anti-cancer, anti-malaria, anti-TB and anti-HIV (Kongkathip & Kongkathip, 2009; Yenjai *et al.*, 2000; Thongthoom *et al.*, 2010) properties. During the course of our on-going research on chemical constituents and bioactive compounds from *Clausena* plants (Maneerat *et al.*, 2010; Maneerat & Laphookhieo, 2010; Sripisut & Laphookhieo, 2010), the title compound (I) which was known as glycozolidal (Li *et al.*, 1991) was isolated from the roots of *C. lansium* which was collected from Nan province in the northern part of Thailand. Herein the isolation and crystal structure of (I) was reported.

In the structure of (I), C_15_H_13_NO_3_ (Fig. 1), the carbazole ring system (C1–C12/N1) is essentially planar with an *r.m.s*. deviation of 0.0093 (1) Å. The aldehyde substituent is planarly attached to the benzene ring which can be indicated by the torsion angle C4–C3–C14–O2 = -3.35 (16)°. whereas the two methoxy groups are slightly deviated from their attached benzene rings with the torsion angles C13–O1–C2–C1 = -6.03 (14)° and C15–O3–C6–C7 = 13.32 (13)°. The bond lengths and angles in (I) are within normal ranges (Allen *et al.*, 1987) and are comparable to the related structures (Fun *et al.*, 2007, 2009, 2010).

In the crystal packing (Fig. 2), N—H···O intermolecular hydrogen bonds (Table 1) connected the molecules into one dimensional chains along the [0 0 1] direction. The crystal is consolidated by short N···O [2.9758 (11) Å] contact, as well as by N—H···O hydrogen bonds, C—H···O and C—H···π (Table 1) and π–π interactions with the distances of *Cg*1···*Cg*1^iv^ = 3.8613 (6) Å *Cg*1···*Cg*2^iv^ = 3.5924 (6) Å and *Cg*2···*Cg*3^iv^ = 3.6576 (6) Å [symmetry code: (iv) 1/2 - *x*, 1/2 - *y*, 1 - *z*; *Cg*1, *Cg*2 and *Cg*3 are the centroids of the C9–C12/N1, C1–C4/C11/C12 and C5–C10 rings, respectively]. .

### Experimental

The air dried roots of *C. lansium* (2.92 kg) were successively extracted with acetone over the period of 3 days at room temperature. The solvent was removed under reduced pressure to provide the acetone extract (61.46 g) which was subjected to quick column chromatography (QCC) over silica gel and eluted with a gradient of hexanes-EtOAc (100% hexane to 100% EtOAc) to provide eight fractions (A—H). Fraction C (14.79 g) was further separated by sephadex LH-20 with CH_3_OH to give six subfractions (C1—6). Subfraction C4 (5.70 g) was subjected to repeated QCC using 20% hexanes-EtOAc yielding the title compound (I) (19.6 mg). Yellow block-shaped single crystals of the title compound suitable for *x*-ray structure determination were recrystallized from CH_2_Cl_2_/acetone (1:4, *v*/*v*) after several days, Mp 469.6–470.7 K.

### Refinement

The H atom attached to N1 was located in a difference map and isotropically refined. The remaining H atoms were placed in calculated positions with *d*(C—H) = 0.95 Å for aromatic and CH, and 0.98 Å for CH_3_ atoms. The *U*_iso_(H) values were constrained to be 1.5*U*_eq_ of the carrier atom for methyl H atoms and 1.2*U*_eq_ for the remaining H atoms. A rotating group model was used for the methyl groups. The highest residual electron density peak is located at 0.69 Å from atom C5 and the deepest hole is located at 0.64 Å from atom C9.

### Crystal data

**Table:**

C_15_H_13_NO_3_	*F*(000) = 1072
*M**_r_* = 255.26	*D*_x_ = 1.461 Mg m^−^^3^
Monoclinic, *C*2/*c*	Melting point = 469.6–470.7 K
Hall symbol: -C 2yc	Mo *K*α radiation, λ = 0.71073 Å
*a* = 20.5756 (4) Å	Cell parameters from 3381 reflections
*b* = 8.1298 (1) Å	θ = 2.0–30.0°
*c* = 14.0411 (3) Å	µ = 0.10 mm^−^^1^
β = 98.871 (1)°	*T* = 100 K
*V* = 2320.64 (7) Å^3^	Block, yellow
*Z* = 8	0.53 × 0.42 × 0.16 mm

### Data collection

**Table:**

Bruker APEXII CCD area-detector diffractometer	3381 independent reflections
Radiation source: sealed tube	3032 reflections with *I* > 2σ(*I*)
graphite	*R*_int_ = 0.020
φ and ω scans	θ_max_ = 30.0°, θ_min_ = 2.0°
Absorption correction: multi-scan (*SADABS*; Bruker, 2005)	*h* = −28→28
*T*_min_ = 0.947, *T*_max_ = 0.984	*k* = −11→9
13003 measured reflections	*l* = −19→15

### Refinement

**Table:**

Refinement on *F*^2^	Primary atom site location: structure-invariant direct methods
Least-squares matrix: full	Secondary atom site location: difference Fourier map
*R*[*F*^2^ > 2σ(*F*^2^)] = 0.039	Hydrogen site location: inferred from neighbouring sites
*wR*(*F*^2^) = 0.115	H atoms treated by a mixture of independent and constrained refinement
*S* = 1.04	*w* = 1/[σ^2^(*F*_o_^2^) + (0.0682*P*)^2^ + 1.6186*P*] where *P* = (*F*_o_^2^ + 2*F*_c_^2^)/3
3381 reflections	(Δ/σ)_max_ = 0.001
178 parameters	Δρ_max_ = 0.45 e Å^−^^3^
0 restraints	Δρ_min_ = −0.24 e Å^−^^3^

### Special details

**Table:**

Experimental. The crystal was placed in the cold stream of an Oxford Cryosystems Cobra open-flow nitrogen cryostat (Cosier & Glazer, 1986) operating at 120.0 (1) K.
Geometry. All e.s.d.'s (except the e.s.d. in the dihedral angle between two l.s. planes) are estimated using the full covariance matrix. The cell e.s.d.'s are taken into account individually in the estimation of e.s.d.'s in distances, angles and torsion angles; correlations between e.s.d.'s in cell parameters are only used when they are defined by crystal symmetry. An approximate (isotropic) treatment of cell e.s.d.'s is used for estimating e.s.d.'s involving l.s. planes.
Refinement. Refinement of *F*^2^ against ALL reflections. The weighted *R*-factor *wR* and goodness of fit *S* are based on *F*^2^, conventional *R*-factors *R* are based on *F*, with *F* set to zero for negative *F*^2^. The threshold expression of *F*^2^ > σ(*F*^2^) is used only for calculating *R*-factors(gt) *etc*. and is not relevant to the choice of reflections for refinement. *R*-factors based on *F*^2^ are statistically about twice as large as those based on *F*, and *R*- factors based on ALL data will be even larger.

### Fractional atomic coordinates and isotropic or equivalent isotropic displacement parameters (Å^2^)

**Table:**

	*x*	*y*	*z*	*U*_iso_*/*U*_eq_	
O1	0.06869 (4)	0.45177 (10)	0.60778 (5)	0.01915 (17)	
O2	0.01915 (4)	0.13634 (10)	0.39162 (6)	0.02408 (18)	
O3	0.34527 (3)	0.26588 (9)	0.16940 (5)	0.01588 (16)	
N1	0.27840 (4)	0.55708 (10)	0.49606 (6)	0.01503 (17)	
H1N1	0.2977 (8)	0.626 (2)	0.5410 (12)	0.031 (4)*	
C1	0.17456 (5)	0.51795 (12)	0.56255 (7)	0.01507 (18)	
H1A	0.1855	0.5922	0.6148	0.018*	
C2	0.11513 (5)	0.43366 (12)	0.54868 (7)	0.01486 (19)	
C3	0.09900 (5)	0.32215 (12)	0.47030 (7)	0.01479 (18)	
C4	0.14322 (5)	0.29659 (12)	0.40535 (7)	0.01420 (18)	
H4A	0.1325	0.2221	0.3532	0.017*	
C5	0.27380 (4)	0.30870 (12)	0.28279 (7)	0.01392 (18)	
H5A	0.2440	0.2330	0.2479	0.017*	
C6	0.33378 (5)	0.34504 (12)	0.25284 (7)	0.01366 (18)	
C7	0.37905 (5)	0.45348 (12)	0.30457 (7)	0.01510 (18)	
H7A	0.4196	0.4759	0.2826	0.018*	
C8	0.36473 (5)	0.52877 (12)	0.38837 (7)	0.01506 (19)	
H8A	0.3955	0.6003	0.4248	0.018*	
C9	0.30431 (5)	0.49645 (12)	0.41708 (7)	0.01365 (18)	
C10	0.25861 (4)	0.38624 (11)	0.36532 (6)	0.01310 (18)	
C11	0.20281 (4)	0.38018 (11)	0.41712 (6)	0.01329 (18)	
C12	0.21752 (4)	0.48914 (11)	0.49667 (7)	0.01357 (18)	
C13	0.08690 (5)	0.55135 (13)	0.69203 (7)	0.0199 (2)	
H13A	0.0510	0.5519	0.7305	0.030*	
H13B	0.0955	0.6641	0.6726	0.030*	
H13C	0.1266	0.5061	0.7306	0.030*	
C14	0.03699 (5)	0.23074 (13)	0.45831 (8)	0.0192 (2)	
H14A	0.0089	0.2457	0.5053	0.023*	
C15	0.41126 (5)	0.27000 (14)	0.14839 (8)	0.0200 (2)	
H15A	0.4153	0.1932	0.0958	0.030*	
H15B	0.4419	0.2380	0.2059	0.030*	
H15C	0.4217	0.3816	0.1291	0.030*	

### Atomic displacement parameters (Å^2^)

**Table:**

	*U*^11^	*U*^22^	*U*^33^	*U*^12^	*U*^13^	*U*^23^
O1	0.0164 (3)	0.0238 (4)	0.0186 (4)	−0.0010 (3)	0.0070 (3)	−0.0029 (3)
O2	0.0178 (4)	0.0251 (4)	0.0294 (4)	−0.0050 (3)	0.0038 (3)	−0.0061 (3)
O3	0.0146 (3)	0.0201 (3)	0.0135 (3)	0.0005 (2)	0.0042 (2)	−0.0016 (2)
N1	0.0141 (4)	0.0167 (4)	0.0145 (4)	−0.0030 (3)	0.0028 (3)	−0.0026 (3)
C1	0.0157 (4)	0.0159 (4)	0.0137 (4)	−0.0003 (3)	0.0025 (3)	−0.0008 (3)
C2	0.0142 (4)	0.0162 (4)	0.0147 (4)	0.0011 (3)	0.0039 (3)	0.0015 (3)
C3	0.0135 (4)	0.0156 (4)	0.0154 (4)	−0.0007 (3)	0.0025 (3)	0.0012 (3)
C4	0.0144 (4)	0.0145 (4)	0.0136 (4)	−0.0010 (3)	0.0017 (3)	0.0007 (3)
C5	0.0139 (4)	0.0143 (4)	0.0134 (4)	−0.0007 (3)	0.0018 (3)	0.0004 (3)
C6	0.0149 (4)	0.0145 (4)	0.0118 (4)	0.0013 (3)	0.0029 (3)	0.0010 (3)
C7	0.0133 (4)	0.0164 (4)	0.0157 (4)	−0.0014 (3)	0.0029 (3)	0.0016 (3)
C8	0.0143 (4)	0.0156 (4)	0.0152 (4)	−0.0026 (3)	0.0021 (3)	0.0004 (3)
C9	0.0138 (4)	0.0137 (4)	0.0133 (4)	−0.0004 (3)	0.0018 (3)	0.0006 (3)
C10	0.0129 (4)	0.0138 (4)	0.0127 (4)	−0.0006 (3)	0.0021 (3)	0.0014 (3)
C11	0.0134 (4)	0.0143 (4)	0.0123 (4)	−0.0004 (3)	0.0024 (3)	0.0008 (3)
C12	0.0132 (4)	0.0140 (4)	0.0133 (4)	−0.0003 (3)	0.0015 (3)	0.0012 (3)
C13	0.0226 (5)	0.0206 (5)	0.0178 (5)	0.0012 (4)	0.0074 (4)	−0.0015 (4)
C14	0.0143 (4)	0.0206 (5)	0.0232 (5)	−0.0019 (3)	0.0047 (3)	−0.0002 (4)
C15	0.0166 (4)	0.0243 (5)	0.0204 (5)	−0.0012 (4)	0.0073 (4)	−0.0016 (4)

### Geometric parameters (Å, °)

**Table:**

O1—C2	1.3667 (11)	C5—C10	1.3962 (13)
O1—C13	1.4347 (13)	C5—H5A	0.9500
O2—C14	1.2225 (13)	C6—C7	1.4013 (13)
O3—C6	1.3887 (11)	C7—C8	1.3975 (13)
O3—C15	1.4337 (11)	C7—H7A	0.9500
N1—C12	1.3702 (11)	C8—C9	1.3896 (13)
N1—C9	1.3927 (12)	C8—H8A	0.9500
N1—H1N1	0.890 (17)	C9—C10	1.4158 (13)
C1—C2	1.3890 (13)	C10—C11	1.4516 (12)
C1—C12	1.3944 (13)	C11—C12	1.4212 (13)
C1—H1A	0.9500	C13—H13A	0.9800
C2—C3	1.4250 (13)	C13—H13B	0.9800
C3—C4	1.3995 (13)	C13—H13C	0.9800
C3—C14	1.4637 (13)	C14—H14A	0.9500
C4—C11	1.3892 (13)	C15—H15A	0.9800
C4—H4A	0.9500	C15—H15B	0.9800
C5—C6	1.3954 (12)	C15—H15C	0.9800
			
C2—O1—C13	116.31 (8)	C7—C8—H8A	120.8
C6—O3—C15	116.91 (8)	C8—C9—N1	129.31 (9)
C12—N1—C9	108.96 (8)	C8—C9—C10	121.59 (9)
C12—N1—H1N1	123.8 (11)	N1—C9—C10	109.10 (8)
C9—N1—H1N1	127.2 (11)	C5—C10—C9	119.72 (8)
C2—C1—C12	117.29 (9)	C5—C10—C11	134.11 (8)
C2—C1—H1A	121.4	C9—C10—C11	106.16 (8)
C12—C1—H1A	121.4	C4—C11—C12	118.40 (8)
O1—C2—C1	122.99 (9)	C4—C11—C10	135.13 (9)
O1—C2—C3	115.86 (8)	C12—C11—C10	106.47 (8)
C1—C2—C3	121.15 (9)	N1—C12—C1	127.59 (9)
C4—C3—C2	120.03 (9)	N1—C12—C11	109.30 (8)
C4—C3—C14	119.50 (9)	C1—C12—C11	123.10 (9)
C2—C3—C14	120.45 (9)	O1—C13—H13A	109.5
C11—C4—C3	120.02 (9)	O1—C13—H13B	109.5
C11—C4—H4A	120.0	H13A—C13—H13B	109.5
C3—C4—H4A	120.0	O1—C13—H13C	109.5
C6—C5—C10	118.43 (8)	H13A—C13—H13C	109.5
C6—C5—H5A	120.8	H13B—C13—H13C	109.5
C10—C5—H5A	120.8	O2—C14—C3	124.07 (10)
O3—C6—C5	115.45 (8)	O2—C14—H14A	118.0
O3—C6—C7	122.88 (8)	C3—C14—H14A	118.0
C5—C6—C7	121.67 (9)	O3—C15—H15A	109.5
C8—C7—C6	120.15 (8)	O3—C15—H15B	109.5
C8—C7—H7A	119.9	H15A—C15—H15B	109.5
C6—C7—H7A	119.9	O3—C15—H15C	109.5
C9—C8—C7	118.40 (9)	H15A—C15—H15C	109.5
C9—C8—H8A	120.8	H15B—C15—H15C	109.5
			
C13—O1—C2—C1	−6.03 (14)	C6—C5—C10—C11	179.98 (10)
C13—O1—C2—C3	174.23 (8)	C8—C9—C10—C5	−0.65 (14)
C12—C1—C2—O1	−179.61 (9)	N1—C9—C10—C5	−179.76 (8)
C12—C1—C2—C3	0.12 (14)	C8—C9—C10—C11	178.56 (9)
O1—C2—C3—C4	179.41 (8)	N1—C9—C10—C11	−0.55 (10)
C1—C2—C3—C4	−0.33 (14)	C3—C4—C11—C12	0.77 (14)
O1—C2—C3—C14	−2.02 (14)	C3—C4—C11—C10	−179.17 (10)
C1—C2—C3—C14	178.23 (9)	C5—C10—C11—C4	−0.18 (19)
C2—C3—C4—C11	−0.13 (14)	C9—C10—C11—C4	−179.22 (10)
C14—C3—C4—C11	−178.71 (9)	C5—C10—C11—C12	179.87 (10)
C15—O3—C6—C5	−166.26 (8)	C9—C10—C11—C12	0.83 (10)
C15—O3—C6—C7	13.32 (13)	C9—N1—C12—C1	−179.26 (9)
C10—C5—C6—O3	−178.94 (8)	C9—N1—C12—C11	0.49 (11)
C10—C5—C6—C7	1.47 (14)	C2—C1—C12—N1	−179.72 (9)
O3—C6—C7—C8	−179.69 (8)	C2—C1—C12—C11	0.56 (14)
C5—C6—C7—C8	−0.13 (14)	C4—C11—C12—N1	179.22 (8)
C6—C7—C8—C9	−1.58 (14)	C10—C11—C12—N1	−0.82 (10)
C7—C8—C9—N1	−179.11 (9)	C4—C11—C12—C1	−1.01 (14)
C7—C8—C9—C10	1.98 (14)	C10—C11—C12—C1	178.95 (9)
C12—N1—C9—C8	−178.97 (10)	C4—C3—C14—O2	−3.35 (16)
C12—N1—C9—C10	0.05 (11)	C2—C3—C14—O2	178.08 (10)
C6—C5—C10—C9	−1.08 (13)		

### Hydrogen-bond geometry (Å, °)

**Table:**

*Cg*2 is the centroid of the C1–C4/C11/C12 ring.

**Table:**

*D*—H···*A*	*D*—H	H···*A*	*D*···*A*	*D*—H···*A*
N1—H1N1···O3^i^	0.890 (17)	2.106 (17)	2.9758 (11)	165.2 (15)
C15—H15C···O2^ii^	0.98	2.44	3.3888 (14)	162
C15—H15A···Cg2^iii^	0.98	2.91	3.6613 (12)	134

Symmetry codes: (i) *x*, −*y*+1, *z*+1/2; (ii) −*x*+1/2, *y*+1/2, −*z*+1/2; (iii) −*x*+1/2, *y*−1/2, −*z*+1/2.
